# Hamiltonian dynamics of the SIS epidemic model with stochastic fluctuations

**DOI:** 10.1038/s41598-019-52351-x

**Published:** 2019-11-01

**Authors:** Gilberto M. Nakamura, Alexandre S. Martinez

**Affiliations:** 1 Faculdade de Filosofia, Ciências e Letras de Ribeirão Preto (FFCLRP), Universidade de São Paulo, Avenida Bandeirantes 3900, 14040-901 Ribeirão Preto, Brazil; 2 Instituto Nacional de Ciência e Tecnologia - Sistemas Complexos (INCT-SC), 22460-320 Rio de Janeiro, Brazil; 3 Present Address: Laboratoire d’Imagerie et Modélisation en Neurobiologie et Cancérologie (IMNC), Centre National de la Recherche Scientifique (CNRS), UMR 8165, Universités Paris 11 and Paris 7, Campus d’Orsay, 91405 Orsay, France

**Keywords:** Applied mathematics, Biological physics, Statistical physics

## Abstract

Empirical records of epidemics reveal that fluctuations are important factors for the spread and prevalence of infectious diseases. The exact manner in which fluctuations affect spreading dynamics remains poorly known. Recent analytical and numerical studies have demonstrated that improved differential equations for mean and variance of infected individuals reproduce certain regimes of the SIS epidemic model. Here, we show they form a dynamical system that follows Hamilton’s equations, which allow us to understand the role of fluctuations and their effects on epidemics. Our findings show the Hamiltonian is a constant of motion for large population sizes. For small populations, finite size effects break the temporal symmetry and induce a power-law decay of the Hamiltonian near the outbreak onset, with a parameter-free exponent. Away from the onset, the Hamiltonian decays exponentially according to a constant relaxation time, which we propose as a metric when fluctuations cannot be neglected.

## Introduction

Models of disease transmission, or epidemic models for short, have been an integral part of the epidemiological toolkit, dating back from pioneer models of Kermack and McKendrick^1^. The main goal of epidemic models can be summarized as the ability to accurately predict spreading patterns of a given communicable disease afflicting a specific population. These models allow decision makers to assess the various intervention strategies available to them and to plan accordingly. Several approaches have been developed to model disease outbreaks^2^, namely, compartmental equations, stochastic equations, agent-based simulations, etc. Each approach suits a particular aspect of the outbreak being studied, built upon hypotheses compatible with empirical records or based on a phenomenological context. They include, but are not restricted to, biological content of the disease, mechanisms behind pathogen transmission, social interactions among the target population and its spatial structure^3^. By the same token, different models for the same disease and population may produce inconsistent results, possibly due to conflicting underlying hypotheses. For instance, the random-mixing hypothesis–i.e. the population is assumed to be homogeneous and its elements mix at random–seems reasonable to model pathogen transmission for airborne disease like influenza, but it seems equivocated for sexually transmitted diseases^4^. Recent studies using household data shows that the random-mixing hypothesis can produce reliable predictions for households, even for heterogeneous contact networks^5^. In general, available empiric data and numerical simulations provide evidence that the disease spreading is largely affected by the heterogeneity of contact network of the population^6–12^.

Despite the significant advances obtained in the past few decades, several challenges remain open. One issue concerns the failure to account for effects unrelated to diseases themselves, such as vaccination skepticism, which ultimately reduces children immunization rate. Outbreaks of treatable communicable diseases, like measles, are on the rise^13^. Another issue deals with understanding the complex dynamics and processes behind infections in both small and large scales^14–16^. To put it simply, there are too many variables and their effects are not entirely known due to the non-linear nature of the problem. As a consequence, the full extent of variable changes or their fluctuations remains poorly understood, which may produce sub-optimal intervention strategies. As an example, detailed field data from the recent Ebola epidemic have shown that smaller outbreaks from different localities are asynchronous^17^. The lack of synchronization between different populations reduces the likelihood of pathogen eradication on a global scale, as long as migration is allowed in some form^18,19^. The effects of migration and spatial structures in epidemic models and pathogen variability have been under investigation for some time^20,21^, and they have been linked to chaotic dynamics in local population^22^. Experiments on the effects of migration between metapopulations, i.e. similar populations but spatially separated, subjected to temporal fluctuations have shown that pathogen prevalence is greatly influenced by the nature of the fluctuation^23^, highlighting the interplay between synchronization and pathogen prevalence in epidemics^24^.

Traditionally, the detailed examination of fluctuations–either temporal or spatial–and their effects on system dynamics have been largely described by correlation functions^23,25^. More recently, autocorrelation functions have been used to reveal the nature and general aspects of fluctuations in a simple agent-based epidemic model for a population of size *N*, in which temporal fluctuations are divided into two broad classes: gaussian and non-gaussian^26^. In the gaussian regime, the prevalence of the disease is well described by its instantaneous average, finite variance, and higher cumulants can be neglected. This is remarkable as it allows one to derive the exact contributions of fluctuations to disease outbreaks in the asymptotic limit *N* $\gg$ 1. Here, we show that the dynamical equations form a Hamiltonian dynamical system, and the way external noise can be incorporated to model disease outbreaks. This approach allows us to discuss quantitatively the relevant scales of the problem, and interpret the resulting Lagrangian and canonical transformations.

## Model

**Figure 1:**
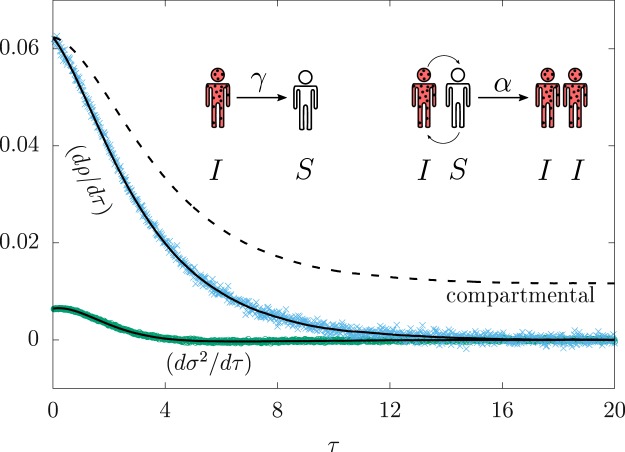
Numerical simulations of the SIS model. (inset) Infected hosts (I) recover to susceptible state (S) with rate *γ* (left). The adequate interaction between an infected host with a susceptible one may trigger a new infection, with rate *α* (right). Stochastic effects are far more relevant for small population sizes (*N* = 50, *γ*/*α* = 1/2), reducing the accuracy of compartmental equations. The forward-derivative *dρ*/*dτ* from data (cross) agrees with Eq. (5a) (solid line), while the compartmental equation Eq. (1) fails to reproduce the data (dashed line). The forward-derivative *dσ*^2^/*dτ* from data (circles) also agrees with the formula in Eq. (5b) (line). All the lines are drawn using the simulated data for 〈*ρ*(*τ*)〉, *σ*^2^(*τ*), and Δ_3_(*τ*).

We begin our discussion using the susceptible-infectious-susceptible (SIS) epidemic model. The SIS model describes the dissemination of a single communicable disease in a susceptible population of size *N*. The transmission of the pathogen occurs when infectious hosts transmit the disease pathogen to healthy susceptible individuals. The infectious period extends throughout the whole course of the disease until the recovery of the patient, warranting a two-stage model: either infected or susceptible. The essence of the model is summarized by inset in Fig. 1.

The traditional formulation of the problem assumes the random-mixing hypothesis (see Introduction) holds for a large population size *N*$\gg$1, compromised of statistically equivalent individuals. Under these circumstances, the only relevant variable is the instantaneous density of infected elements *ρ*(*t*), which means that fluctuations can be safely neglected. Furthermore, *ρ*(*t*) decreases with rate *γρ*, where *γ* is the recovery rate. New infections per unit of time (disease incidence) are proportional to *αρ*(1 − *ρ*), i.e., they depend on the chance that infected elements interact with susceptible ones, with intensity given by the transmission rate *α*. This picture provides an interpretation where *ρ*(*t*) is continuously exchanged between two compartments, leading a simple description called compartmental equation: *dρ*(*t*)/*dt* = *αρ*(1 − *ρ*) − *γρ*. For the sake of convenience, redefine the timescale as *τ* ≡ *αt* and *ρ*_0_ ≡ 1 − *γ*/*α*, so that

1 $$\frac{d}{d\tau }\rho (\tau )=\rho ({\rho }_{0}-\rho \mathrm{)}.$$

Clearly, the equilibrium density can either be *ρ*_*eq*_ = 0 or *ρ*_*eq*_ = *ρ*_0_. Also, *ρ*_0_ is related to the basic reproduction number *R*_0_ = *N*(*α*/*γ*) which provides an estimate on the number of new infections per generation^27^.

In light of its long age, compartmental equations have met considerable success in predicting the time evolution of disease outbreaks, providing valuable insights for intervention strategies and funding allocation^28^. However, outbreaks that fail to meet the underlying hypotheses (random mixing and large population of statistically equivalent elements) can contradict compartmental equations. These inconsistencies are largely attributed to stochastic effects and their inherent fluctuations^2^.

## Improved Compartmental Equations

Stochastic variables are known to cause the emergence of critical phenomena in computer simulations of epidemic models, under certain parameter ranges^29,30^. One key ingredient common to critical phenomena is the scale invariance of fluctuations. This special symmetry remains the foundation of cooperative phenomena and critical phase transitions, whose contributions span over a broad set of research fields such as condensed-matter, quantum field theories, and neuroscience to name a few^31–36^. In these systems, fluctuations occur in all sizes and dictate the general behavior of the problem, which prompts for in-depth studies of their effects in epidemic models.

Stochastic epidemic models include non-deterministic events that intrinsically occurs during the course of the disease spreading process. Examples of these events include the transmission of the pathogen, and the elapsed time required for the complete recovery of patients. The inclusion of these effects brings the models closer to more realistic expectations, which in turn can deviate from predictions using compartmental equations^37–39^. One prime example is the effects of the absorbing state in the SIS model, which has been examined in detail by Nåsell and others^40–42^. New experiments on this subject provide evidence that temporal fluctuations can drastically alter the prevalence of pathogens^23^. Spatial heterogeneity also introduces an extra layer of complexity as it may trap or delay the pathogen transmission^20^. As a result, the requirements of statistical equivalence may not hold for all scales. To deal with this issue, stochastic formulations and numerical simulations have been the default tools to investigate fluctuations in disease outbreaks. In what follows, we outline the general ideas behind the agent-based approach to describe the SIS model. A more detailed account of the results listed here are explained in detail in refs^26,43,44^.

Our discussion assumes the disease spreading follows a Markov chain in discrete time *δt*. Moreover, *δt* is such that at most a single recovery or transmission event is likely to occur during the course of its duration. Under these requirements, the master equation of the SIS model in discrete time reads

2 $$\frac{d{P}_{\mu }(t)}{dt}=-\,\mathop{\sum }\limits_{\nu =0}^{{2}^{N}-1}\,{H}_{\mu \nu }{P}_{\nu }(t\mathrm{)}.$$

Here, *P*_*μ*_(*t*) refers to the instantaneous probability to observe the system in the *μ*-th configuration. Configuration labels follow the binary ruling $\mu ={n}_{0}{2}^{0}+{n}_{1}{2}^{1}+\cdots +{n}_{N-1}{2}^{N-1}$, where *n*_*k*_ = 1 if the *k*-th agent is infected, or *n*_*k*_ = 0 otherwise, with *k* = 0, 1, …, *N* − 1. For instance, for *N* = 3, the configuration |*μ* = 3〉 = |110〉 states that only the agent with label *k* = 2 is susceptible. The matrix elements *H*_*μν*_ express the transition rates from configuration *ν* to configuration *μ*. By virtue of probability conservation, in each time step the transition rules satisfy ${\sum }_{\mu }{H}_{\mu \nu }\,=\,0$. The matrix elements ${H}_{\mu \nu }=\langle \mu |\hat{H}|\nu \rangle$ are computed from projections on the time step operator

3 $$\hat{H}=\frac{\alpha }{N}\mathop{\sum }\limits_{k,\ell =0}^{N-1}\,{A}_{k\ell }(1-{\hat{n}}_{k}-{\hat{\sigma }}_{k}^{+}){\hat{n}}_{\ell }+\gamma \mathop{\sum }\limits_{k=0}^{N-1}\,({\hat{n}}_{k}-{\hat{\sigma }}_{k}^{-}),$$

where ${A}_{k\ell }$ is the adjacency matrix, ${\hat{n}}_{k}$ represents the *k*-th occupation operator (with eigenvalues *n*_*k*_ = 1 if infected, 0 otherwise), and ${\hat{\sigma }}_{k}^{+}$ are the localized spin-1/2 ladder operators that produce the transition *S* → *I*. Clearly, ${\hat{\sigma }}_{k}^{-}$ produce the opposite transitions, *I* → *S* in relation the *k*-th agent. As notation, the hat symbol always accompanies operators to quickly distinguish them from numbers.

The master equation Eq. (2) provides the means to evaluate the time evolution of relevant statistical moments of *ρ*(*t*). Notice that the average density of infected agents in the system reads

4 $$\langle \rho (t)\rangle =\frac{1}{N}\mathop{\sum }\limits_{\mu =0}^{{2}^{N}-1}\,\mathop{\sum }\limits_{k=0}^{N-1}\,\langle \mu |{\hat{n}}_{k}|\mu \rangle {P}_{\mu }(t).$$

Applying the time derivative, and using Eq. (2), one arrives at the equation of motion for 〈*ρ*(*t*)〉. Useful expressions are known only for a few types of adjacency matrix *A*. The simplest one is the complete graph ${A}_{k\ell }\,=\,1-{\delta }_{k\ell }$, which recovers the random mixing hypothesis. In those particular instances, the complete time evolution of the system comprehends a set of hierarchical equations that involves the statistical moments of *ρ*(*t*), as shown in refs. ^43,45^. More explicitly^45^, the first two equations for instantaneous mean 〈*ρ*〉 and variance *σ*^2^ = 〈*ρ*^2^〉 − 〈*ρ*〉^2^ are

5a $$\frac{d\langle \rho \rangle }{d\tau }=\langle \rho \rangle [{\rho }_{0}-\langle \rho \rangle ]-{\sigma }^{2}(\tau ),$$

5b $$\frac{d{\sigma }^{2}}{d\tau }=2{\sigma }^{2}[{\rho }_{0}+\langle \rho \rangle ]-2{\Delta }_{3}(\tau )+\frac{1}{N}\langle \rho (1-\rho )\rangle +\frac{\gamma }{N\alpha }\langle \rho \rangle ,$$

where Δ_3_(*τ*) = 〈*ρ*^3^(*τ*)〉 − 〈*ρ*(*τ*)〉^3^. These results find excellent agreement with simulated data using an ensemble with 10^6^ replicas starting from the same initial condition (see Fig. 1).

Comparing Eqs (1) and (5), the case that considers temporal fluctuations decays faster than the compartmental equation by *σ*^2^(*τ*), even in the regime $N\gg 1$. Both equations are equivalent whenever *σ*(*τ*) becomes irrelevant compared to 〈*ρ*〉. Therefore, a generalization of compartmental equations for the SIS model is readily available by retaining both mean and variance, neglecting higher statistical moments. Thus, the dynamical system describes a gaussian variable evolving along time. The skewness coefficient vanishes as a direct consequence of this assumption, so that Δ_3_(*τ*) ≈ 3〈*ρ*(*τ*)〉*σ*^2^(*τ*). For $N\gg 1$, the resulting equations are

6a $$\frac{d}{d\tau }\,\mathrm{ln}\,\langle \rho \rangle ={\rho }_{0}-\langle \rho \rangle -\frac{{\sigma }^{2}}{\langle \rho \rangle },$$

6b $$\frac{1}{2}\frac{d}{d\tau }\,\mathrm{ln}\,{\sigma }^{2}={\rho }_{0}-2\langle \rho \rangle .$$

We emphasize that the variance in Eq. (6a) slows down the growth rate of 〈*ρ*(*τ*)〉, recalling the Allee effect^28,46^.

The dynamical system represented by Eq. (6) can also be obtained from a stochastic expansion following the guidelines in ref.^47^. In this case, assume the density *ρ* = 〈*ρ*〉 + *η* is well described by its instantaneous average plus some noise function *η*(*τ*). We assume 〈*η*〉 = 0 and 〈*η*^2^〉 = *σ*^2^(*τ*) for the sake of consistency. The additional requirement $|\eta |\ll \langle \rho \rangle$ ensures stochastic effects act as perturbations. Expanding Eq. (1) as a Taylor series around *ρ* = 〈*ρ*〉, and then taking the ensemble average, produces

7 $$\frac{d\langle \rho \rangle }{d\tau }=\langle g(\langle \rho \rangle )+\eta {\frac{dg}{d\rho }|}_{\langle \rho \rangle }+\frac{{\eta }^{2}}{2}{\frac{{d}^{2}g}{d{\rho }^{2}}|}_{\langle \rho \rangle }+o({\eta }^{3})\rangle ,$$

where *g*(*ρ*) = *ρ*(*ρ*_0_ − *ρ*). Since 〈*η*〉 vanishes, the expansion recovers Eq. (6a) up to *o*(*η*^4^). The task to obtain the equation of motion of *σ*^2^ is greatly simplified by noting that

8 $$\eta \frac{d\eta }{dt}=\eta [\frac{d\rho }{dt}-\frac{d\langle \rho \rangle }{dt}]={\eta }^{2}[{\rho }_{0}-2\langle \rho \rangle ]-{\eta }^{3}+\eta \langle {\eta }^{2}\rangle .$$

Taking the ensemble average, one obtains (1/2)(*d*/*dt*)*lnσ*^2^ = [*ρ*_0_ − 2〈*ρ*〉] − 〈*η*^3^〉/*σ*^2^. Now, since *η* mimics a gaussian variable, 〈*η*^3^〉 = 0 and Eq. (6b) is recovered as well.

Equation (6) can be further combined into a single second-order differential equation^26^, with solution

9a $$\langle \rho (\tau )\rangle =\frac{{\rho }_{0}(1+{c}_{1}{{\rm{e}}}^{-{\rho }_{0}\tau })}{1+2{c}_{1}{{\rm{e}}}^{-{\rho }_{0}\tau }+{c}_{2}{{\rm{e}}}^{-2{\rho }_{0}\tau }},$$

9b $${\sigma }^{2}(\tau )=\frac{{\langle \rho (\tau )\rangle }^{2}({c}_{1}^{2}-{c}_{2}){{\rm{e}}}^{-2{\rho }_{0}\tau }}{{(1+{c}_{1}{{\rm{e}}}^{-{\rho }_{0}\tau })}^{2}}.$$

The constants *c*_1_ and *c*_2_ depend solely on the initial conditions. The special case ${c}_{2}={c}_{1}^{2}$ recovers the usual solution of Eq. (1). We assumed that fluctuations behave as gaussian fluctuations. While reasonable for various situations, the assumption does not hold for *γ*/*α* around unity or small population sizes, according to numerical simulations^26^, in which Eq. (5b) should be used instead of Eq. (6b). For the sake of completeness, there is another solution in which *σ*^2^(*τ*) = 〈*ρ*(*τ*)〉^2^, with$\langle \rho (\tau )\rangle ={\rho }_{0}/(2+{c}_{1}{e}^{-{\rho }_{0}\tau })$.

## Hamilton Equations

The fact that the dynamical system Eq. (6) can be combined into a single second-order differential equation suggests an interpretation of the epidemic model in terms of Hamilton equations^48^. Hamiltonian systems are ubiquitous in Physics, serving as basis to describe and explain countless physical phenomena. The hallmark of systems are the Hamilton equations:

10a $$\frac{dq}{d\tau }=\,\frac{\partial  {\mathcal H} }{\partial p},$$

10b $$\frac{dp}{d\tau }=-\frac{\partial  {\mathcal H} }{\partial q},$$

where *q*(*t*) and *p*(*t*) are conjugated variables, and the Hamiltonian function ${\mathcal H}$ encodes some information about the problem–usually associated with energy for conservative systems but not restricted to them. Besides classical mechanics and related areas, quantum field theories and statistical mechanics are deeply intertwined with Hamilton’s principle and Liouville theorem. Despite its usefulness in Physics, Hamilton formulation and surrounding principles are rarely used in population dynamics, ecological problems, or epidemic models, where first-order differential equations are dominant. The lack of second-order differential equations in these areas, although not prohibitive, raises questions about the description of the dynamics, as discussed extensively in ref. ^49^. In part, because it means some interactions and stochastic effects acting on the system remain unaccounted. Significant advances in the Hamiltonian formulation of stochastic epidemic models have been obtained using the eikonal approximation, with emphasis on the disease extinction and vaccination^50,51^. Generalizations for heterogeneous networks have also been investigated, providing improved control strategies^52,53^. Even more, the aforementioned Hamiltonian formulation explains other effects, such as the emergence of noise-induced metastable states^54^. The crucial ingredient of these studies concerns the eikonal approximation, in which either the generating function or the density of infected is written as the exponential of the classical action of the system^55^. For populations with fixed size, one variable describes 〈*ρ*(*t*)〉, while its conjugate variable encompasses fluctuations^52^. However, the description of epidemics using this conjugate pair remains a complex task because the conjugate variable is not directly related to familiar statistics such as the standard deviation or variance. In what follows, instead of using the eikonal approach, we argue that Hamilton dynamics for the stochastic SIS model can be constructed from 〈*ρ*(*τ*)〉 and *σ*(*τ*).

In view of the inherent stochasticity behind disease spreading, it seems necessary to determine whether Eq. (6) form a Hamiltonian system or not. A brief inspection shows the pair (〈*ρ*〉, *σ*^2^) does not satisfy the usual Hamilton equations. The solution to this issue is obtained by assuming, instead, that the correct conjugated pair is (〈*ρ*〉, *h*(*σ*^2^)), where *h*(*x*) is some analytical function. Inspiration from common pairs of conjugate variables can be used to refine the choice of *h*(*x*). For instance, the product 〈*ρ*〉 × *h*(*σ*^2^) should be dimensionless, in close analogy the scalar product between position and wave vectors. One possible candidate is *h*(*x*) = *x*^−1/2^, which entails 1/*σ* as the conjugated variable to 〈*ρ*〉.

Define the dynamical variables *q*(*τ*) = 〈*ρ*(*τ*)〉 and *p*(*τ*) = 1/*σ*(*τ*) to describe the SIS model. In addition, consider the following Hamiltonian

11 $$ {\mathcal H} =q(\tau )p(\tau )[{\rho }_{0}-q(\tau )]+\frac{1}{p(\tau )}.$$

Plugging these expressions in Eq. (10), one obtains the equations of motion:

12a $$\frac{dq}{d\tau }=q({\rho }_{0}-q)-\frac{1}{{p}^{2}}\equiv \langle \rho \rangle \,[{\rho }_{0}-\langle \rho \rangle ]-{\sigma }^{2},$$

12b $$\frac{dp}{d\tau }=-p({\rho }_{0}-2q)\equiv -\,\frac{1}{\sigma }[{\rho }_{0}-2\langle \rho \rangle ].$$

Thus, at first glance ${\mathcal H}$ appears to be a valid candidate to describe the SIS model. Even more, replacing (*q*, *p*) by Eq. (9) in Eq. (11) shows that the Hamiltonian is a constant of motion ${ {\mathcal H} }^{\infty }={\rho }_{0}{c}_{1}{({c}_{1}^{2}-{c}_{2})}^{-1/2}$. The upper index in ${ {\mathcal H} }^{\infty }$ is a reminder that calculations take place in the absence of finite size corrections.

**Figure 2:**
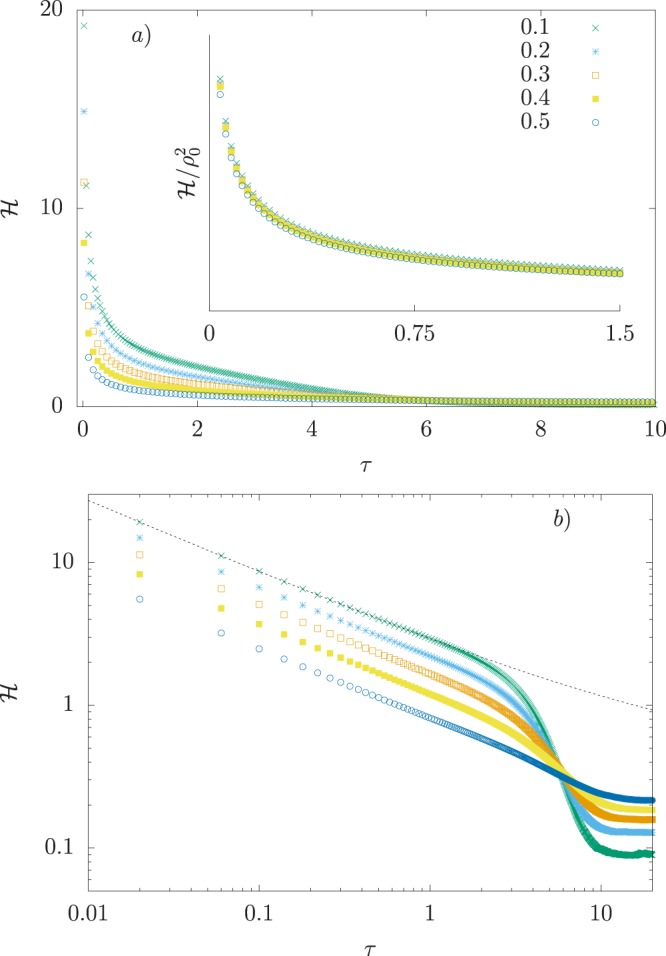
Finite size effects on the Hamiltonian. (**a**) Simulated data with *N* = 50 and 10^6^ Monte Carlo runs for various ratios *γ*/*α*. (inset) Data collapse using the scaling factor *ρ*_0_^2^, suggesting an universal behavior at the beginning of the outbreak. (**b**) Initial decay of ${\mathcal H}$ compatible with power-law, ${\mathcal H}  \sim {\tau }^{-\lambda }$. The exponent *λ* = 1/2 remains constant for different ratios *γ*/*α*, suggesting an universal behavior.

However, taking finite size corrections into account changes drastically the notion of ${\mathcal H}$ as a constant of motion. In fact, as Fig. 2 depicts, numerical simulations for finite populations reveal ${\mathcal H}$ changes continuously along time until equilibrium sets in, akin to a non-conservative system. The precise meaning of ${\mathcal H}$ in the epidemiological context is still murky, at best. A detailed analysis of correlations between changes in ${\mathcal H}$ and the spreading pattern of real outbreaks is mandatory to understand the action-reaction analogy. In the meantime, it is instructive to study ${\mathcal H}$ for *τ* *≪* 1 and *τ* *≫* 1 (see Fig. 2). For *τ* *≪* 1, where incidentally fluctuations vary the most (see Fig. 1), a remarkable feature appears via the relation ${\mathcal H}  \sim {\tau }^{-\lambda }$ with *λ* = 1/2. In particular, the exponent *λ* seems insensitive to changes in the epidemiological parameter *γ*. This parameter-free behavior is not observed for the remaining statistics, 〈*ρ*(*τ*)〉 and *σ*(*τ*). Power-laws are crucial to identify scaling relations and the emergence of universal features, and they are usually related to the symmetry of the problem rather than microscopic details. Here, evidence of universal behavior is captured by the data collapse ${\mathcal H} /{\rho }_{0}^{2}$ (Fig. 2a inset). From these observations, we can infer fluctuations play a larger role in the early disease spreading, being largely independent of exact values of epidemiological parameters.

An effective decay ${e}^{-\tau /{\tau }_{eff}}$ describes the general behavior of ${\mathcal H}$ away from the outbreak onset. The relaxation time *τ*_*eff*_ depends on *N* and the ratio *γ*/*α*, and it can be estimated from data by fitting ${\mathcal H}$ to an exponential function plus a constant. Alternatively, it can be evaluated as

13 $${\tau }_{eff}=\frac{1}{ {\mathcal H} (0)}{\int }_{0}^{\infty }\,d\tau [ {\mathcal H} (\tau )- {\mathcal H} (\infty )].$$

From a formal point of view, the evaluation of *τ*_*eff*_ requires the solutions of Eqs (5a) and (5b) in Eq. (11), followed by an integration. Surely, the procedure is arguably more demanding than estimating *R*_0_. However, as others have reasoned before, *R*_0_ provides a naive estimation on secondary infections because the growth rate of the outbreak changes continuously along time^56^. In contrast, *τ*_*eff*_ mimics a constant of motion.

## Lagrangian and Canonical Transformations

Another insight from *τ*_*eff*_ links the temporal integral of ${\mathcal H}$ with the mechanical action *S*. A formal connection with *S* is desirable because it brings a large machinery revolving around variational principles and conservation laws. However, the action $S=\int \,d\tau  {\mathcal L} (q,\dot{q};\tau )$ is a functional of the Lagrangian ${\mathcal L}$. It turns out that ${\mathcal L}$ can obtained from ${\mathcal H}$ by inspection. From Eqs (15) and (7), ${\mathcal H}$ takes the following form: ${\mathcal H} =p[q({\rho }_{0}-q)+{p}^{-2}]=p(dq/d\tau )+2/p$. Recalling the formal expression ${\mathcal H} =p\dot{q}- {\mathcal L}$, it becomes clear that

14 $$ {\mathcal L} =-\,\frac{2}{p}=-\,2\sigma (\tau )=-\,2\sqrt{q({\rho }_{0}-q)-\frac{dq}{d\tau }},$$

where we have used Eq. (6a) and considered only the positive root. Thus, ${\mathcal L}$ is proportional to the standard deviation while the action entails the accumulated deviation over the course of the outbreak. To check our result for large populations $N\gg 1$, the minimal action recovers Eq. (1) as expected for a noise-free system. In general, the equation of motion reads

15 $$\frac{{d}^{2}q}{d{\tau }^{2}}=3({\rho }_{0}-2q)[\frac{dq}{d\tau }-\frac{2}{3}q({\rho }_{0}-q)].$$

The fact that ${\mathcal L}$ contains solely the standard deviation allows us to understand how to add uncorrelated fluctuations into the model. By virtue of *Var*[*x* + *y*] = *Var*[*x*] + *Var*[*y*] for uncorrelated random variables *x* and *y*, the perturbed Lagrangian can be obtained by adding a *σ*_*ext*_^2^(*τ*) to the variance of the system *σ*^2^(*τ*):

16 $$ {\mathcal L} \text{'}=-\,2\sqrt{q({\rho }_{0}-q)-\frac{dq}{d\tau }+{\sigma }_{ext}^{2}(\tau )}.$$

This picture is consistent with addition of a noise function *σ*_*ext*_^2^(*τ*) to Eq. (6a). The perturbed Lagrangian ${\mathcal L} \text{'}$ describes, ultimately, the time evolution of the disease prevalence in environments with noise. Note that this description differs from the usual derivation of Langevin equations, in which the noise function (force) *r*(*τ*) couples linearly with *q*, i.e., ${\mathcal L} \text{'}= {\mathcal L} -r(\tau )q(\tau )$. By the same token, the addition of correlated signals *η*(*τ*) to the Lagrangian entails corrections from the covariance matrix: since *Var*[*X* + *Y*] = *Var*[*X*] + *Var*[*Y*] + 2*Cov*[*X*, *Y*], then ${\mathcal L} \text{'}=-\,2\sqrt{{\sigma }_{\rho }^{2}+{\sigma }_{\eta }^{2}+2{\rm{Cov}}[\rho ,\eta ]}$. The covariance matrix can estimated or modeled directly from data, promoting further understanding on the spreading of co-existing diseases, where facilitation or competition processes are in place.

With both Hamiltonian and Lagrangian formalisms secured, canonical transformations become available. These transformations are particularly useful to highlight properties of the dynamical systems and to solve them. They change the old variables (*q*, *p*) into new variables (*Q*, *P*), while preserving Hamilton’s equations. There are a large number of transformations available: it would render impossible to cover all of them here. Instead, we show that at least one canonical transformation exists, and that it promotes the interpretation of the stochastic spreading process as effective mechanical systems. Consider: *P*_1_(*t*) = 2*p*^1/2^*q* and *Q*_1_(*t*) = −*p*^1/2^. The Poisson bracket {*Q*_1_, *P*_1_}_*q*,*p*_ = (∂*Q*_1_/∂*q*)(∂*P*_1_/∂*p*) − (∂*Q*_1_/∂*p*)(∂*P*_1_/∂*q*) = 1 shows the transformation is canonical. Setting *m* = 2, the Hamiltonian in terms of the canonical variables (*Q*_1_, *P*_1_) becomes

17 $$-{ {\mathcal H} }_{1}=\frac{1}{2m}{({P}_{1}+{\rho }_{0}{Q}_{1})}^{2}-\frac{{\rho }_{0}^{2}{Q}_{1}^{2}}{2m}-\frac{1}{{Q}_{1}^{2}}.$$

One may interpret $-{ {\mathcal H} }_{1}$ as the Hamiltonian of an effective mechanical problem in one-dimension, in which the particle has mechanical momentum *P*_1_(*τ*), with generalized coordinate *Q*_1_(*τ*), subjected to a velocity dependent potential.

## Conclusion

The description of several real-world problems often includes stochastic fluctuations. The SIS epidemic model includes them due to uncertainties associated with pathogen transmission. For small fluctuation amplitudes, 〈*ρ*(*τ*)〉 and *σ*^2^(*τ*) are adequate descriptors. Our findings demonstrate 〈*ρ*(*τ*)〉 and 1/*σ*(*τ*) are conjugated variables, and they satisfy Hamilton’s equation. These results link the stochastic SIS epidemic model with a pure dynamical system, which can be solved and manipulated using standard analytical tools. We find the Hamiltonian is a constant of motion for *N* $\gg$ 1. However, finite size effects break the temporal symmetry of the system: ${\mathcal H}  \sim {\tau }^{-\mathrm{1/2}}$ follows a power-law around the outbreak onset. A clear explanation for this scaling is still lacking. The relaxation time *τ*_*eff*_ portrays the decay of ${\mathcal H}$ until equilibrium sets in, meaning that it can also be used to characterize the SIS epidemic. Unlike popular estimates of epidemic growth rate, *τ*_*eff*_ remains constant along time and can be extracted from data values of ${\mathcal H}$. Finally, our results also suggest a way to incorporate interactions into the SIS model via the Lagrangian function. This finding has intriguing implications for our understanding of facilitation-competition mechanisms between co-occurring diseases since it does not replicate the canonical procedure to obtain Langevin equations.

## Data Availability

Numerical codes, simulation data and data descriptors are available at 10.17605/OSF.IO/WFCEP.
